# Gender differences in risk factors for ischemic stroke: a longitudinal cohort study in East China

**DOI:** 10.1186/s12883-024-03678-0

**Published:** 2024-05-23

**Authors:** Xinping Bai, Zifeng Li, Zhuo Cai, Mingren Yao, Lin Chen, Youmeng Wang

**Affiliations:** 1 Department of Neurology, Fuyang People’s Hospital, Anhui, 236000 People’s Republic of China; 2 Department of Neurology, Fuyang Hospital Affiliated to Bengbu Medical University, Anhui, 236000 People’s Republic of China

**Keywords:** Stroke, Prevalence, Risk factors, Gender, Roc analysis

## Abstract

**Objectives:**

Epidemiological studies of stroke and its risk factors can help develop strategies to prevent stroke. We aimed to explore the current gender-specific prevalence of stroke and associated risk factors.

**Methods:**

Data were collected using a structured precoded questionnaire designed by the Stroke Screening and Prevention Programme of the National Health and Wellness Commission Stroke Prevention and Control Project Committee, between June 2020 and November 2021. A total of 7394 residents took part in the study, 187 of whom had a stroke. The baseline information of each participant was obtained and included in this study. The chi-square test and Kruskal-Wallis tests were used to examine the relationship between these indicators and stroke, and then multivariate logistic regression was used to construct the prediction scale between different genders.

**Results:**

of 7394 participants,4571 (61.82%) were female. The overall prevalence of stroke patients in the study population was 2.53%, Multivariate analysis found that residence status (OR = 0.43, *p* = 0.002) 、HCY (OR = 0.962, *p* = 0.000)、Previous TIA (OR = 0.200, *p* = 0.002) 、Hypertension (OR = 0.33, *p* = 0.000) and Dyslipidemia (OR = 0.668, *p* = 0.028) were significant predictors of stroke. there are gender differences in the traditional risk factors for stroke, and women have more risk factors. ROC analysis confirmed the accuracy of the stroke risk model, and the AUC of the stroke risk model for the general population was 0.79 with *p* < 0.05. In the gender model, the female AUC was 0.796 (*p* < 0.05). and the male AUC was 0.786 with *p* < 0.05.

**Conclusion:**

The prevalence of stroke in adults aged 40 years and above is high in eastern China were high. management of risk factors can effectively prevent the occurrence of most strokes. more attention should be paid to gender differences associated with stroke.

## Introduction

stroke is a leading cause of death and disability, making its prevention a global health priority [1, 2], Although the incidence of stroke has remained stable over the past 20 years, disability-adjusted life years lost to stroke and the number of stroke-related survivors and deaths are increasing. accounting for 10% [3] of all deaths and 5% [4] of all disability-adjusted life-years worldwide, posing a serious threat to population health, particularly in low - or middle-income developing countries [5]. China faces the greatest challenge from stroke with the highest stroke incidence and mortality in the world [6] and currently accounts for nearly 30% of the total number of stroke-related deaths worldwide [7]. According to the 2019 Global Burden of Disease data, China has the highest lifetime risk of stroke at 39.3%, which means 118 million people in China will suffer from stroke by 2030 [8]. A predictive study using Global Burden of Disease(GBD)2016 data found that the lifetime risk of stroke in people aged 25 years and older is close to 25%, and this will be a major concern for the prevention and management of stroke in the future [8].

The key to the primary prevention of stroke is to adopt reliable individualized risk assessment tools, accurately identify high-risk individuals of stroke in the population, and carry out targeted prevention interventions and risk factor management, to reduce or delay the occurrence of stroke. Some studies have built stroke risk prediction models based on traditional risk factors such as age, systolic blood pressure, diabetes, lipid levels, family history of stroke (FHS), obesity, atrial fibrillation (AF), and lifestyle behaviors (e g, smoking, high alcohol consumption) [9–11]. This model can identify high-risk groups only when the risk factors have changed, which cannot meet the need to carry out preventive intervention as soon as possible. However, few cohort studies have assessed associations between socioeconomic status (different age groups, geographic and economic areas, education level, living situation, marital status, and occupation), lifestyle behaviors (plant-based or meat diets, and consumption of vegetables and fruits), and stroke. To meet the need for further prevention of “pass forward”. Notably, the INTERSTROKE study results revealed that the management of risk factors can effectively prevent the occurrence of most strokes [12].

Interestingly, there are large regional variations in stroke epidemiology in China. The incidence and prevalence of stroke in China show a north-to-south gradient, and the burden of stroke is heaviest in the central region. The epidemiological survey of stroke at the national level is of great significance for the national formulation of public health policies, rational allocation of limited medical resources, and establishment of an effective prevention and control system. However, due to the large geographical span in China, the epidemiological characteristics of stroke are significantly different among regions. Therefore, it is important to reveal the epidemiological characteristics of stroke and related risk factors in the region for the development of appropriate prevention and control strategies. Therefore, we investigated stroke-related risk factors and epidemic characteristics in Fuyang City, East China’s Anhui Province.

Gender studies on stroke incidence vary from country to country, with reports of higher rates in men than women, higher rates in women than men, or no difference between the two. The results of the National Stroke Epidemiology Survey showed that there was no statistically significant gender difference in stroke incidence and age-standardized incidence. Based on the results of 18 studies (including the United States, Japan, Russia, Italy, Finland, etc.), scholars concluded [13] that the incidence of stroke in females was 1.24 times that of males (1.09–1.42). Recent data from Canada shows that over the life cycle, women have a higher risk of ischemic stroke and transient ischemic attack (TIA) than men I [12, 14]. The global estimated lifetime risk of stroke from age 25 onward was 24.7% (23.3–26.0) in men and 25.1% (23.7–26.5) in women. but there is geographical variation [8]. Approximately 55,000 more fatal strokes occur in women each year than men [15]. About 55,000 more fatal strokes occur in women than men each year. For the first time in the Greater Cincinnati Northern Kentucky Stroke Study (GCNKSS) in 2015, stroke case fatality rates for women exceeded those for men, even after adjusting for age [16, 17]. Throughout adulthood, stroke accounts for a higher percentage of deaths in women than in men. However, due to the different reproductive statuses of women, the incidence and significance of stroke risk factors in women are different from those in men. High blood pressure, atrial fibrillation, diabetes, abdominal obesity, migraine with aura, emotional stress, and depression are more common risk factors in women than men. Consideration of sex-specific risk factors can improve individualized stroke risk assessment.

In this study, we determined the distribution of stroke in Chinese adults in 2020–2021 and assessed risk factors for stroke. In addition, we separately assessed risk factors for stroke by gender to provide evidence for future improvements in primary stroke prevention.

## Materials and methods

### Study design and participants

This is a large population-based study conducted by the China National Stroke Screening Survey (CNSSS) established by the National Health and Wellness Commission Stroke Prevention and Control Project Committee [18, 19], The study subjects were community residents in Fuyang who had lived there for some time between June 2020 and November 2021 (6 months or more in the past 12 months). The two-stage stratified cluster sampling method was used for screening. In the first phase, a structured face-to-face questionnaire was used to screen for basic demographic information (sex, age, marital status, education level, social health care, living status, number of siblings or children), stroke history, and exposure to major stroke risk factors before the screening year. In the second stage, the residents with a history of stroke and high-risk groups were investigated by trained neurologists to understand the type, clinical symptoms, diagnosis time, frequency of incidence, history of related chronic diseases and corresponding treatment, and other possible influencing factors of stroke. Venous blood was collected from participants who fasted for 8 h. Laboratory-related index data were obtained by rapid detection in the laboratory by medical personnel. All interview data is electronically entered into a data terminal with direct access to the CNSSI database.

### Measurement of risk factors

Smoking, for this study, was defined as continuous or cumulative smoking for ≥ 6 months in one’s lifetime, having smoked within the 30 days prior to the survey, and currently smoking at least one cigarette per day at the time of the survey [20]. Current alcohol consumption was defined as consuming at least one drink per week [21]. Height and weight measurements were taken with respondents removing their shoes and hats, and BMI was calculated by dividing weight (kg) by the square of height (m). According to the report of the Chinese Obesity Working Group [22], a BMI of ≥ 24 kg/m2 was classified as obese. Blood pressure was measured using an upper-arm electronic sphygmomanometer, with each participant undergoing two measurements after resting for at least 20 min. Hypertension was defined as a mean systolic blood pressure of ≥ 140 mmHg, a mean diastolic blood pressure of ≥ 90 mmHg, or the use of antihypertensive medication within the past two weeks [23]. Diabetes mellitus was defined as a fasting glucose level of ≥ 7.0 mmol/L or a previous diagnosis of diabetes mellitus with the use of glucose-lowering drugs in the past two weeks. Dyslipidemia was defined as a serum TC of ≥ 6.22 mmol/L, a TG of ≥ 2.26 mmol/L, HDL-C of < 1.04 mmol/L, LDL-C of ≥ 4.14 mmol/L, or the use of lipid-lowering drugs.

### Statistical analysis

The IBM SPSS Statistics for Windows, version 27.0 (IBM Corp., Armonk, NY, USA) software package was used for the statistical analysis of the data. The baseline characteristics of all participants were statistically described and divided into two groups based on stroke occurrence. The demographic and clinical characteristics of the two groups were compared. The data that followed a normal or an approximately normal distribution are expressed by mean ± standard deviation, and those that did not follow a normal distribution are expressed in quartiles [P50 (P25, P75)]. When comparing two groups of measurement data, We calculated means and medians to describe continuous variables. We tested the statistical differences using the Mann–Whitney U-test for skew-distributed continuous variables, the t-test for normally distributed continuous variables, and the Chi-square test for categorical variables. Multivariate Logistic regression analysis was performed to determine independent risk factors. Multiple. In addition, we separately analyzed the related factors of stroke in male and female groups and constructed corresponding risk prediction scales. Receiver operating characteristic (ROC) analysis was used to verify the accuracy of the prediction scale. All statistical tests were bilateral tests, P<. 0.05 was considered statistically significant.

## Results

General socio-demographic characteristics of the study population.

Table 1 details the demographic characteristics of the study participants. Of the 7394 participants,4571 (61.82%) were female. The overall prevalence of stroke patients in the study population was 2.53%, There were no significant differences between groups in terms of gender (χ2 = 0.73, *p* > 0.05) and age. In addition, we found that There were significant differences between groups in terms of education, Education Level, residence status, and dietary behaviors (*P* < 0.05). Personal history of chronic diseases such as hypertension, diabetes, dyslipidemia, previous TIA, and atrial fibrillation were statistically significant in the comparison between the two groups.

**Table 1:** Comparison of general information about the study population

Demographic and clinical data	Stroke patients(*n* = 187)	Controls(*n* = 7207)	χ^2^ / z	*P*-value
Age(years)	58(48 ~ 69)	57(50 ~ 69)	-0.799	0.424
Gender (n (%))				
male	77(41.2%)	2746(38.1%)	0.73	0.393
female	110(58.8%)	4461(61.9%)		
Residence (n (%))				
urban	144(77.7%)	3901(54.1%)	38.5	<0.001
Rural	43(23.0%)	3306(45.9%)		
Education Level (n (%))			21.119	<0.001
Primary school and below	78(41.7%)	3188(44.2%)		
Junior Middle School	56(29.9%)	2851(39.6%)		
Senior Middle School	37(19.8%)	782(10.9%)		
College and above	16(8.6%)	386(5.3%)		
Marital status (n (%))			0.171	0.679
Married	174(93.0%)	6647(92.2%)		
Unmarried or widowed	13(7.0%)	560(7.8%)		
Average annual income (n (%))			14.488	0.002
Less than 5,000￥	60(32.1%)	2092(29.0%)		
5,000￥-10,000￥	24(12.8%)	1469(20.4%)		
10,000￥-20,000￥	21(11.2%)	1209(16.8%)		
20,000￥ or more	82(43.9%)	2437(33.8%)		
Residence status (n (%))			0.561	0.454
Living alone	11(5.9%)	339(4.7%)		
Living with others	176(94.1%)	6868(95.3%)		
Taste (n (%))			28.276	<0.001
Salty	53(28.3%)	1092(15.2%)		
Oily	40(21.4%)	2393(33.2%)		
Prefer sweet food	94(50.3%)	3722(51.6%)		
Vegetarian (n (%))			20.074	<0.001
Balanced meat and vegetables	20(10.7%)	400(5.6%)		
Eat more meat	52(27.8%)	3024(42.0%)		
Eat mainly vegetarian food	115(61.5%)	3783(52.5%)		
Vegetables consumption (n (%))			19.523	<0.001
≥5 days/week	127(67.9%)	3868(53.7%)		
3–4 days/week	41(21.9%)	2718(37.7%)		
≤2 days/week	19(10.2%)	621(8.6%)		
Fruit consumption (n (%))			1.046	0.593
≥5days/week	39(20.9%)	1518(21.1%)		
3-4days/week	50(26.7%)	2156(29.9%)		
≤2 days/week	98(52.4%)	3533(49.0%)		
Smoking (n (%))	33(17.6%)	684(9.5%)	13.847	<0.001
Alcohol consumption (n (%))			0.357	0.837
No drinking habits	147(78.6%)	5655(78.5%)		
Drinking regularly	35(18.7%)	1402(19.5%)		
Alcohol consumption	5(2.7%)	150(2.1%)		
BMI(kg/m2)	24.78(23.29 ~ 27.96)	24.54(22.97 ~ 26.75)	-1.676	0.094
FBG (mmol/L)	4.7(3.9 ~ 5.4)	4.6(4 ~ 5.3)	-0.239	0.811
Glycated hemoglobin()	5.3(4.9 ~ 5.6)	5.2(4.8 ~ 5.6)	-0.913	0.361
TC (mmol/L)	4.51(3.92 ~ 5.43	4.83(4.15 ~ 5.61)	-3.084	0.002
TG (mmol/L)	1.54(1.07 ~ 2.13)	1.42(1.03 ~ 2.04)	-0.799	0.424
LDL-C (mmol/L)	2.44(1.75 ~ 3.08)	2.64(2.04 ~ 3.27)	-2.614	0.009
HDL-C (mmol/L)	1.36(1.17 ~ 1.63)	1.42(1.21 ~ 1.68)	-2.603	0.009
HCY(mmol/L)	17.2(12.8 z 22.23)	14.3(10.8 ~ 19.1)	-5.148	<0.001
Family history of stroke (n (%))	21(11.2%)	545(7.6%)	3.469	0.063
Previous TIA (n (%))	5(2.7%)	31(0.4%)	18.938	<0.001
Hypertension (n (%))	150(80.2%)	3610(50.1%)	66.179	<0.001
Diabetes (n (%))	41(21.9%)	872(12.1%)	16.3	<0.001
Dyslipidemia (n (%))	102(55.1%)	2800(38.9%)	20.131	<0.001
Overweight or obese (n (%))	43(23.0%)	1074(14.9%)	9.307	0.002
Lack of exercise (n (%))	51(%)	1601(%)	2.688	0.101
Atrial fibrillation or valve disease (n (%))	3(1.6%)	30(0.4%)	5.79	0.016

BMI, body mass index;; FBG, fasting blood glucose; HDL-C, high density lipoprotein cholesterol; LDL-C,

low density lipoprotein cholesterol; TC, total cholesterol; TG, triglycerides; HCY, homocysteine

Factors associated with Stroke.

Variables with *p* < 0.05 in the univariate analysis were selected for multivariate analysis. The results showed that residence status (OR = 0.430, *p* = 0.002) 、HCY (OR = 0.962, *p* = 0.000)、Previous TIA (OR = 0.200, *p* = 0.002) 、Hypertension (OR = 0.33, *p* = 0.000) and Dyslipidemia (OR = 0.668, *p* = 0.028) were significant predictors of stroke. as shown in Table 2.

**Table 2:** Multivariate logistic regression analysis for stroke

Variables	B	*P*-value	0R(95%CI)
Age	0.006	0.453	1.006(0.991 ~ 1.021)
Gender			
male	1		
female	-0.026	0.894	0.974(0.665 ~ 1.427)
Residence			
urban	-0.844	0.002	0.430(0.250 ~ 0.741)
Rural	1		
Education Level			
Primary school and below	1	0.203	
Junior Middle School	0.246	0.262	1.279(0.832 ~ 1.964)
Senior Middle School	-0.255	0.344	0.775(0.457 ~ 1.314)
College and above	-0.078	0.825	0.925(0.464 ~ 1.843)
Average annual income			
Less than 5,000￥	1	0.633	
5,000￥-10,000￥	0.098	0.743	1.103(0.615 ~ 1.977)
10,000￥-20,000￥	0.307	0.305	1.359(0.757 ~ 2.441)
20,000￥ or more	-0.049	0.829	0.952(0.607 ~ 1.492)
Taste			
Salty	1	0.128	
Oily	-0.421	0.058	0.656(0.425 ~ 1.015)
Prefer sweet food	-0.022	0.930	0.978(0.603 ~ 1.588)
Vegetarian			
Balanced meat and vegetables	1	0.280	
Eat more meat	-0.036	0.905	0.964(0.531 ~ 1.751)
Eat mainly vegetarian food	0.340	0.141	1.405(0.893 ~ 2.211)
Vegetables consumption			
≥5 days/week	1	0.521	
3–4 days/week	0.141	0.630	1.151(0.649 ~ 2.04)
≤2 days/week	0.327	0.291	1.386(0.756 ~ 2.541)
Smoking	0.276	0.370	1.317(0.721 ~ 2.406)
TC	0.306	0.104	1.359(0.939 ~ 1.966)
LDL-C	-0.123	0.536	0.884(0.599 ~ 1.306)
HDL-C	-0.173	0.515	0.841(0.499 ~ 1.417)
HCY	-0.036	0.000	0.965(0.949 ~ 0.981)
Previous	-1.612	0.002	0.2(0.072 ~ 0.555)
Hypertension	-1.108	0.000	0.33(0.22 ~ 0.495)
Diabetes	-0.158	0.441	0.853(0.571 ~ 1.277)
Dyslipidemia	-0.404	0.028	0.668(0.466 ~ 0.957)
Overweight or obese	-0.223	0.445	0.8(0.4;1 ~ 1.419)
Atrial fibrillation or valve disease	-1.165	0.069	0.312(0.089 ~ 1.094)

Gender differences in risk factors for ischemic stroke.

we separately assessed stroke risk factors by gender and the results are shown in Table 3. Variables with *p* < 0.05 in the univariate analysis were selected for multivariate analysis. The male population revealed that residence, Oily Taste, Previous TIA, Hypertension, Atrial fibrillation, or valve disease were found to be statistically significant (*P* < 0.05), and for the female population, Education Level, Salty and sweet food Taste, Vegetarian, HCY, Previous TIA, Hypertension, Overweight or obese were found to be statistically significant (*P* < 0.05).

**Table 3:** Gender differences in risk factors for ischemic stroke

Variables	B	*P*-value	0R(95%CI)
**male**			
age	0.000	0.983	1.000(0.979 ~ 1.022)
Residence			
Rural	1		
urban	-0.757	0.026	0.469(0.241 ~ 0.914)
Education Level			
Primary school and below	1.000	0.105	
Junior Middle School	-0.107	0.730	0.899(0.489 ~ 1.651)
Senior Middle School	-0.589	0.103	0.555(0.273 ~ 1.127)
College and above	0.721	0.279	2.056(0.558 ~ 7.577)
Taste			
Salty	1	0.110	
Oily	0.836	0.037	2.308(1.054 ~ 5.056)
Prefer sweet food	0.328	0.290	1.389(0.755 ~ 2.554)
Vegetarian			
Balanced meat and vegetables	1	0.390	
Eat more meat	0.153	0.707	1.165(0.526 ~ 2.582)
Eat mainly vegetarian food	0.473	0.204	1.605(0.774 ~ 3.328)
Smoking	-0.161	0.551	0.852(0.502 ~ 1.444)
HDL-C	0.553	0.102	1.739(0.895 ~ 3.379)
HCY	-0.035	0.003	0.966(0.944 ~ 0.988)
Previous TIA	-1.406	0.082	0.245(0.05 ~ 1.198)
Hypertension	-1.082	0.000	0.339(0.186 ~ 0.618)
Dyslipidemia	-0.388	0.120	0.679(0.416 ~ 1.107)
Atrial fibrillation or valve disease	-2.284	0.005	0.102(0.021 ~ 0.506)
**female**			
age	0.004	0.660	1.004(0.985 ~ 1.025)
Residence			
Rural	1		
urban	-0.130	0.718	0.878(0.434 ~ 1.779)
Education Level			
Primary school and below	1	0.019	
Junior Middle School	0.607	0.045	1.835(1.015 ~ 3.317)
Senior Middle School	0.123	0.754	1.131(0.523 ~ 2.449)
College and above	-0.533	0.207	0.587(0.256 ~ 1.344)
Average annual income			
Less than 5,000￥	1	0.311	
5,000￥-10,000￥	0.164	0.673	1.178(0.551 ~ 2.517)
10,000￥;-20,000￥	0.566	0.208	1.76(0.73 ~ 4.246)
20,000￥ or more	-0.240	0.437	0.786(0.429 ~ 1.441)
Taste			
Salty	1	0.036	
Oily	0.386	0.240	1.472(0.772 ~ 2.804)
Prefer sweet food	0.764	0.010	2.148(1.2 ~ 3.846)
Vegetarian			
Balanced meat and vegetables	1	0.043	
Eat more meat	0.538	0.267	1.712(0.662 ~ 4.427)
Eat mainly vegetarian food	-0.225	0.636	0.798(0.314 ~ 2.028)
Vegetables consumption			
≥5 days/week	1	0.124	
3–4 days/week	0.568	0.074	1.765(0.947 ~ 3.29)
≤2 days/week	-0.178	0.658	0.837(0.38 ~ 1.843)
TC	0.013	0.953	1.014(0.645 ~ 1.592)
LDL-C	0.324	0.207	1.383(0.836 ~ 2.29)
HCY	-0.035	0.002	0.966(0.945 ~ 0.988)
Previous TIA	-2.028	0.003	0.132(0.034 ~ 0.513)
Hypertension	-1.097	0.000	0.334(0.2 ~ 0.557)
Dyslipidemia	-0.398	0.075	0.672(0.433 ~ 1.042)
Overweight or obese	-0.504	0.039	0.604(0.374 ~ 0.976)
Atrial fibrillation or valve disease	-0.227	0.840	0.797(0.088 ~ 7.245)

HDL-C, high-density lipoprotein cholesterol; LDL-C, low-density lipoprotein cholesterol; TC, total cholesterol; HCY, homocysteine

ROC analysis verified the accuracy of stroke risk prediction models for different genders.

**Fig. 1:**
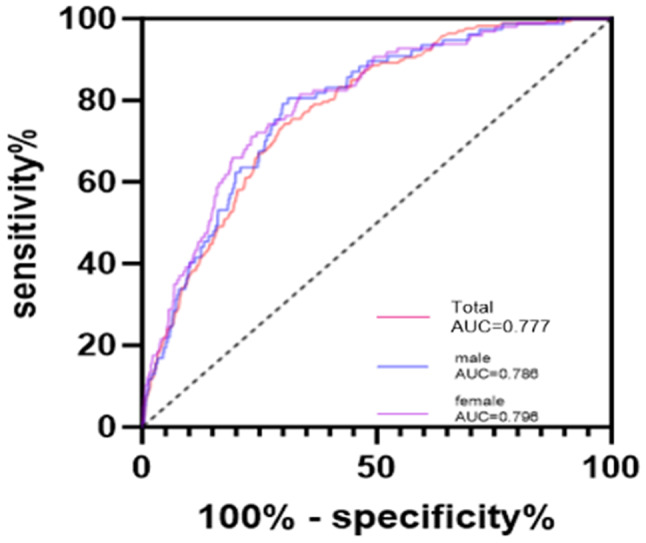
Receiver operating characteristic (ROC) generated for predicting stroke. The stroke prediction model for the total population, the AUC was 0.79, with *p* < 0.05. as for Prediction model by gender, the female AUC was 0.796, with *p* < 0.05 and the male AUC was 0.786, with *p* < 0.05

As shown in Fig. 1. The overall population stroke prediction model AUC was 0.777 (*p* < 0.05). as for the Prediction model by gender, the female AUC was 0.796, with *p* < 0.05, and the male AUC was 0.786, with *p* < 0.05. The results indicated that the three prediction scales could effectively and accurately predict the stroke risk of community residents.

## Discussion

This study adopts multi-stage stratified random cluster sampling to cover the sample population aged 40 and above from 42 villages and 13 cities in Fuyang, Anhui Province. The results of this survey show that the total complex weighted prevalence rate of stroke in Fuyang, from 2020 to 2021 is 2.53%. This is higher than the 2013 China Stroke Prevention Project (CSPP) survey (2.08%).In this survey, the top three risk factors in the prevalence of stroke in Fuyang were hypertension, dyslipidemia, overweight or obesity, and further multivariate logistic regression also found that hypertension, previous TIA, dyslipidemia, and elevated HCY were statistically significant independent risk factors for stroke in this area.

According to China Stroke Prevention and Treatment Report 2020, the regional characteristics of stroke in China are higher in the north and lower in the south, and the characteristics of urban and rural areas are higher than urban areas, which is not only reflected in the incidence, The prevalence rate and mortality rate are also higher in rural areas than in urban areas [24]. In this study, the incidence of stroke in urban communities was higher than that in rural communities (77.7%vs23.0%). which may be related to local healthy living behaviors and stroke prevention and control measures. People in urban communities have a higher awareness of disease prevention and control and more physical examinations and are more likely to find some mild stroke lesions from imaging. This suggests that in the prevention and control of stroke, corresponding stroke prevention and control strategies should be formulated according to the situation of different regions [25]. However, foreign studies have also found that the incidence of stroke is lower in rural areas than in urban areas. The results of the stroke registry in Brest, France, show that the incidence of stroke in suburban areas is significantly lower than that in urban centers (incidence ratio = 0.87, 95%CI: 0.77–0.99) [24]. This is also the case in South Asia and India [26, 27].

It was recognized that obese or overweight individuals were at higher risk for various chronic diseases such as stroke, high blood pressure, diabetes, and other diseases [28–30]. This study suggests that overweight or obesity is an independent risk factor for stroke. Meanwhile, we analyzed the relationship between BMI index and the incidence of stroke, and the results showed that BMI is not a risk factor for the increased incidence of stroke, suggesting that if conditions allow, we can consider using multiple obesity indicators, including waist-height ratio、waist circumference and CVAI(Chinese visceral adiposity index.) to comprehensively evaluate obesity. CVAI was an evaluation index of visceral fat calculated based on the age, BMI, waist circumference, triglyceride, and HDL of the Chinese population [31]. Compared with traditional indexes, CVAI could better reflect the visceral fat content of the human body [32]. There is also a significant correlation between obesity and the cause of cardiovascular and cerebrovascular diseases [33, 34].

The study results on the incidence of stroke by gender vary from country to country, with both men being higher than women, and no gender differences or women being higher than men. This study found no statistically significant difference in the incidence of stroke between genders, and the results of the national stroke epidemiological survey showed no statistically significant gender difference in the incidence and age-standardized prevalence of stroke, which was consistent with the results of this study. Some scholars summarized the results of 18 studies (including the United States, Japan, Russia, Italy, Finland, etc.) and concluded that the incidence of stroke in females was 1.24 times that of males (1.09–1.42) [35], which was inconsistent with the results of this study. The reasons for the inconsistency may be due to different countries and regions, and the differences in the life behaviors of men and women have an impact on the research results. In a Framingham heart study [36], the age of onset of the first stroke in women was significantly higher than that in men (75.1 years vs. 71.1 years), the incidence of stroke in men before 85 years was higher than that in women after 85 years, and the lifetime risk of stroke was higher at all ages, which may be related to the longer average life expectancy of women. There was no significant difference in stroke subtypes and stroke severity between different genders [37]. The results of this study suggest that there are gender differences in the traditional and common risk factors for stroke, and women have more risk factors. In terms of general demographic characteristics, there were statistical differences between the two groups in urban and rural areas and education level, while the average annual income level only had an impact on the risk of stroke in women. In terms of general demographic characteristics, there were statistical differences between the two groups in urban and rural areas and education level, while the average annual income level only had an impact on the risk of stroke in women. The relationship between dietary habits and stroke has received increasing attention, and it is an important aspect of stroke prevention. While previous studies have focused on the relationship between dietary patterns and stroke, our study included more detailed eating behaviors including taste, meat and vegetable diet, vegetable and fruit intake, and their relationship between stroke. Dietary habits including taste, balance of meat and vegetable, and consumption of vegetables were statistically significant in female stroke patients (*P* < 0.01), while taste, balance of meat and vegetable and smoking were risk factors in male stroke patients. A healthy plant-based diet had been linked to a lower risk of stroke [38].Smoking had been recognized as a risk factor for various diseases [39]. Studies across different regions and populations had shown a strong correlation between smoking and stroke, with smokers having a much higher risk of stroke compared to non-smokers or quits [40]. In China, based on China’s traditional culture and life characteristics, the number of female smokers is much smaller than that of men.

In terms of complicated chronic diseases, TIA, hypertension, diabetes, dyslipidemia, overweight or obesity were all risk factors for stroke in women (*P* < 0.05), while the differences in TIA, hypertension and dyslipidemia in men were statistically significant. The 2014 AHA/ASA Stroke Prevention Guidelines for Women state that women with diabetes are at greater risk of stroke than men [41]. A systematic meta-analysis of 64 cohort studies with a total of 775,385 individuals and 12,539 stroke events showed that the maximum adjusted relative risk RR for diabetes-related stroke was 2.28 for women and 1.83 for men, indicating a greater stroke risk for women with diabetes [42]. Key modifiable risk factors appear to influence risk across genders. The risk of ischemic stroke begins to rise when a woman’s fasting blood sugar level is lower than that of a man, even after adjusting for oral medication or insulin therapy. This suggests that diabetes is more strongly associated with ischemic stroke in women than in men. Stroke risk associated with lipid profiles was subtle and varied between stroke subtypes, but there was no consistent difference between genders. Analysis of blood lipids indicated that total cholesterol and low density lipoprotein levels were statistically significant in female stroke patients, while high density lipoprotein levels were statistically significant in male stroke patients.

In this study, we also found that, although among controllable metabolic risk factors, hypertension was the largest risk factor for ischemic stroke (OR = 0.33; 95%CI=(0.220 ~ 0.495). Some scholars have found that high blood pressure has a lifetime risk of stroke, especially for ischemic stroke [43]. For hypertension, Studies have shown mixed results on gender differences between high blood pressure and stroke. Leening M J et al. confirmed that there was no significant gender difference between elevated systolic blood pressure and stroke risk [44], but there were also studies that showed a stronger association between hypertension and the risk of total stroke and ischemic stroke in women compared with men [45]. Data from a recent large-scale observational study involving 27,542 patients suggest that the systolic blood pressure threshold for stroke risk is lower in women than in men. current approaches to using sex-agnostic targets for lowering elevated BP could benefit from careful reassessment. Further investigations are needed to prospectively determine whether ideal treatment targets for hypertension might indeed be lower for women than for men, before sex-specific targets are integrated into guidelines [46].

Previously, stroke risk prediction models based on traditional risk factors such as gender, age, blood pressure and antihypertensive drug treatment, smoking and diabetes played an important role in the screening of high-risk groups of stroke. Our study included some relevant indicators that were not previously covered, including exercise habits, dietary tastes, intake of fruits and vegetables, meat diet, BMI, etc., to complete a more comprehensive analysis of stroke risk factors. We also built predictive models for gender-specific populations. The AUC of women and men were 0.77 and 0.89, respectively, which had a good predictive effect. A prospective study in Japan, based on cohorts from multiple centers, constructed a risk equation for 10-year ischemic stroke incidence with an AUC of 0.78 [47]. similar to our findings. A Chinese study [48] based on the “Prodiction for Atherosclerotic Cardiovascular Disease Risk in China, China-PAR)” project used Cox proportional risk model to construct a 10-year stroke risk prediction model. The C statistic of China-PAR 10-year stroke risk prediction model was 0.810 (95% CI:0.787–0.833) for female and 0.810((95% CI:0.787–0.833)for male, respectively. And the risk of stroke predicted by the model was close to the actual incidence. Although the pathogenesis is unknown [49]. there are significant sex differences in incidence and stroke-related risk factors. Our study provides evidence for the effect of gender on stroke incidence. predicting 10-year and lifetime stroke risk in Chinese population.

To sum up, with the release of stroke prevention guidelines for women in 2014, more and more attention has been paid to gender differences related to stroke. Compared with men, risk factors including diet, education level and diabetes mellitus have a greater impact on the risk of ischemic stroke in women, while the association between smoking and HCY level and stroke in men may be slightly higher than that in women. However, female sex is not an independent risk factor for poor prognosis of stroke. Although significant progress has been made in examining gender differences in stroke and the specific factors that influence risk and outcomes in women, significant research gaps remain.

There are some limitations to our study. First, we did not distinguish between gender risk factors for ischemic and hemorrhagic stroke and compared the clinical characteristics of stroke patients with different subtypes and genders. Secondly, although the study was for the whole group, We found that there was a gender imbalance in the study population, with far more women than men, which may be related to China’s national conditions. During the survey period, more men worked abroad, resulting in selection bias. In addition, we should follow participants over time. The study also lacked other validation cohorts. We will pay more attention to this in future studies.

## Data Availability

No datasets were generated or analysed during the current study.
